# A light-exploiting insectivorous bat shows no melatonin disruption under lights with different spectra

**DOI:** 10.1098/rsos.221436

**Published:** 2023-03-29

**Authors:** Alicia M. Dimovski, Stephen R. Griffiths, Kerry V. Fanson, Danielle L. Eastick, Kylie A. Robert

**Affiliations:** ^1^ School of Agriculture, Biomedicine & Environment, La Trobe University, Melbourne 3086, Australia; ^2^ Research Centre for Future Landscapes, La Trobe University, Melbourne 3086, Australia

**Keywords:** ALAN, circadian disruption, LED, light pollution, urinary melatonin, microchiroptera

## Abstract

Natural light-dark cycles synchronize an animal's internal clock with environmental conditions. The introduction of artificial light into the night-time environment masks natural light cues and has the potential to disrupt this well-established biological rhythm. Nocturnal animal species, such as bats, are adapted to low light conditions and are therefore among the most vulnerable to the impacts of artificial light at night (ALAN). The behaviour and activity of insectivorous bats is disrupted by short-wavelength artificial light at night, while long-wavelength light is less disruptive. However, the physiological consequences of this lighting have not been investigated. Here, we examine the effect of LEDs with different spectra on urinary melatonin in an insectivorous bat. We collected voluntarily voided urine samples from Gould's wattled bats (*Chalinolobus gouldii*) and measured melatonin–sulfate under ambient night-time conditions (baseline) and under red (*λ*P 630 nm), amber (*λ*P 601 nm), filtered warm white (*λ*P 586 nm) and cool white (*λ*P 457 nm) LEDs. We found no effect of light treatment on melatonin–sulfate irrespective of spectra. Our findings suggest that short-term exposure to LEDs at night do not disrupt circadian physiology in the light-exploiting Gould's wattled bat.

## 1. Introduction

The oscillation of natural light-dark cycles provides crucial photoperiod information required to synchronize an animal's circadian clock with its external environment [1]. Circadian rhythms are entrained by short-wavelength light and play a critical role in regulating behaviour and physiology in wildlife [1,2]. The introduction of artificial light has profoundly altered the night-time environment and masks photoperiod changes [2,3]. Consequently, artificial light at night has been identified as a novel stressor for wildlife and poses a key threat to biodiversity [4,5]. Concern for the impacts of artificial light on biological rhythms in wild animals is growing, especially for species residing near urban areas.

The circadian hormone melatonin communicates photoperiod information to the body [6]. Short-wavelength blue light (459–480 nm) activates the photoreceptors responsible for entraining melatonin rhythms in humans [7,8] and animals [6,9]. Light in this spectral range suppresses melatonin; therefore, peak production occurs during the dark phase [1,10,11]. The duration of the melatonin peak provides a crucial signal for encoding season [10,12] and modulates reproduction, immune function, metabolism and thermoregulation in animals [13]. Across animal taxa, exposure to artificial light at night suppresses the normal increase in melatonin [14].

Artificial lights containing blue wavelengths suppress melatonin more efficiently and to a greater extent than longer wavelengths [15–17]. Standard white light-emitting diodes (LEDs) are particularly harmful to wildlife as they contain a large amount of short-wavelength blue light [18]. However, flexibility in the spectral composition of LEDs provides a benefit over previous lighting technologies. Recent studies suggest long-wavelength LEDs reduce the physiological consequences of artificial light on wildlife (e.g. [17,19]).

Nocturnal animals are particularly vulnerable to artificial light at night since they evolved to be active in low-light environments [20,21]. One such group is bats (Chiroptera), which comprise one-third of nocturnal mammal species [22]. Previous research has focused on the effect of artificial light on bat activity, foraging, drinking and commuting behaviour [23–32]. However, little is known about the physiological impacts (except see [33–36]). Some insectivorous bats exploit artificial lights to capitalize on the increased invertebrate abundance at light sources [24,25,30,37], although this may exacerbate the physiological consequences of nocturnal light exposure. Even short exposure to high intensity light in a dark environment suppresses melatonin in Djungarian hamsters (*Phodopus sungorus*) [38] and alters circadian patterns of activity in Schneider's roundleaf bats (*Hipposideros speoris*) [34,35]*.* Recent behavioural studies (e.g. [26]) suggest long-wavelength red lights are a ‘bat friendly’ lighting option. However, the physiological cost associated with exposure to lights with this spectral composition remains unknown.

Here, we investigate melatonin production in Gould's wattled bats (*Chalinolobus gouldii*) exposed to LED lights with different spectra. The Gould's wattled bat is an Australian insectivorous bat species that commonly forages and roosts in urban environments [39–41]. Previous behavioural studies suggest Gould's wattled bats do not avoid artificial lights [40] and may exploit the high abundance of insects attracted to light sources. Therefore, Gould's wattled bats are an ideal model to study the effect of artificial light at night on physiology. To examine the effect of wavelength on melatonin production, we exposed bats to LEDs with different spectra (red, amber, filtered warm white, standard cool white), and monitored urinary melatonin levels. The LEDs were predicted to influence melatonin production to different degrees. Since melatonin is most sensitive to short wavelengths, we predicted lights with a high proportion of blue wavelengths (cool white LEDs) would suppress melatonin to a greater extent than lights with a small proportion of blue wavelengths (warm white LEDs). We also predicted that lights without blue wavelengths (amber and red LEDs) would be the least disruptive to melatonin production. This information will improve our understanding of the effects of artificial light on bat physiology and evaluate options for ‘bat friendly’ LED lighting.

## 2. Material and methods

### 2.1. Experimental design

To investigate the effect of LEDs with different wavelengths on melatonin production in insectivorous bats, we brought Gould's wattled bats (*Chalinolobus gouldii*) into captivity for the duration of the study. All bats (*n* = 27) were held under ambient night-time conditions (baseline) on the first night of the study. Each bat was randomly allocated to a different experimental lighting treatment on nights two, four and six of the study, with 6–7 bats exposed to each light treatment per night. The order of light treatment was randomized for each individual. Over the course of the study each bat was exposed to three lighting treatments. Voluntarily voided urine samples were collected from bats under baseline and light treatments and analysed for 6-sulfatoxymelatonin (melatonin–sulfate), the major melatonin metabolite in urine [42,43]. Bats were left undisturbed and held under ambient night-time conditions between lighting treatments on nights three and five of the study to mitigate any lag effect of light exposure on circadian rhythms.

### 2.2. Animals

This study was conducted during May 2021. Twenty-seven (14 female, 13 male) wild Gould's wattled bats were collected from roost boxes located in the Nangak Tamboree Wildlife Sanctuary (Bundoora, Victoria, Australia) and housed in the La Trobe University Zoology Reserve (Bundoora, Victoria, Australia). Some individuals were already marked with a microchip or band as part of a long-term mark and recapture program [44]. New individuals were banded with a metal-alloy bat-band (Australian Bird and Bat Banding Scheme) for unique identification. Bats were housed in metal bird aviaries (2 m L × 1 m W × 1.85 m H) fitted with a roost box to provide shelter throughout the study. On sampling nights, bats were captured from roost boxes prior to sunset and placed in calico bags for ease of urine collection (detailed below). Each aviary was separated by wooden and metal panels to prevent light trespass between enclosures. Animals were hand-fed mealworms and water until satiated before the start of the active period on sampling nights. Food and water were also provided ad libitum in housing enclosures.

### 2.3. Experimental illumination

**Figure 1.**
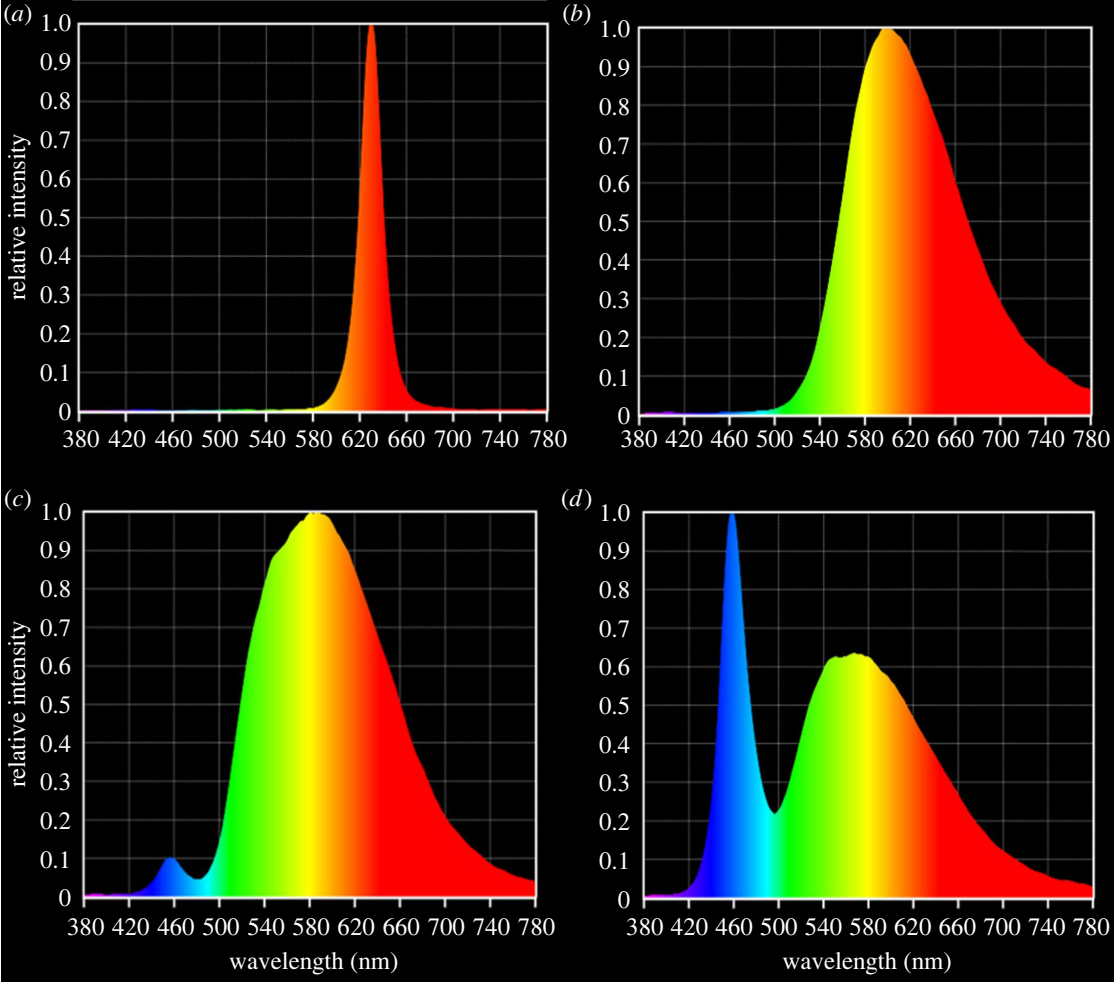
Experimental LED spectra; (*a*) red (*λ*P 630 nm), (*b*) amber (*λ*P 601 nm), (*c*) filtered warm white (*λ*P 586 nm) and (*d*) cool white (*λ*P 457 nm).

Experimental LEDs were suspended on the roof, 2.05 m directly above calico bags (see S2 for experimental enclosure image) to ensure bats were exposed to 10 lux (range 9.98–10.07 lux) inside calico bags, corresponding to the highest intensity standard (PP1) for pedestrian area lighting in urban parklands in Australia (AS/NZS 11:58.3.1:2020). Light fittings consisted of red (*λ*P 630 nm), amber (*λ*P 601 nm), filtered warm white (*λ*P 586 nm) and cool white (*λ*P 457 nm) LEDs (figure 1). All lights were supplied by Hi-Lux Technical Services Pty Ltd. (Thomastown, Victoria, Australia). Lights were switched on at sunset (times obtained from the Australian Bureau of Meteorology (http://www.bom.gov.au)) and switched off once urine samples were collected from all individuals (233–461 min after sunset). A lux meter (EA33 EasyViewTM Light Meter with Memory, Extech, New Hampshire, USA) was used to determine light intensity inside calico bags 1.25–2.25 h after sunset in each experimental enclosure. The spectral composition of experimental lighting was quantified using a hand-held spectrometer (Lighting passport, Asensetek, New Taioei, Taiwan; spectral range 380–780 nm; 5–50 000 lux).

### 2.4. Urine sample collection

To monitor nocturnal urinary melatonin–sulfate concentrations in response to experimental lighting, we collected urine samples from bats under ambient night-time (baseline; *n* = 3 male, 5 female) and under red (*n* = 5 male, 4 female), amber (*n* = 4 male, 5 female), warm white (*n* = 9 male, 3 female) and cool white LEDs (*n* = 6 male, 5 female). On sampling nights, bats were placed in individual calico bags at sunset (times obtained from the Australian Bureau of Meteorology (http://www.bom.gov.au)) and suspended under experimental LEDs. All sampling was conducted between 3.9 and 7.7 h after sunset. During sampling, bats were removed from calico bags and voluntarily voided urine was collected. Bats typically urinated upon handling; if required, massage was used to encourage urination. Some samples were too small (less than 100 µl) for hormone analysis, consequently final sample sizes are lower than the total number of animals used in the study. Following collection, urine samples were stored at −20°C until analysis.

### 2.5. Urinary melatonin–sulfate assay

To investigate the effect of light spectra on melatonin production, we measured 6-sulfatoxymelatonin (melatonin–sulfate), the major melatonin metabolite in urine. The timing and rhythm of urinary melatonin–sulfate concentrations consistently correspond with melatonin in plasma and saliva [45]. A commercial enzyme-linked immunosorbent assay (Cat no. RE54031; IBL International, Hamburg, Germany) was used to measure 6-sulfatoxymelatonin (melatonin–sulfate) in urine samples. The assay was run according to the manufacturer's protocol, except the assay buffer was replaced with Trizma buffer. The assay was biochemically validated in our laboratories by demonstrating parallelism between serial dilutions of a pooled urine sample and the standard curve. Plates were read at 450 nm (620 nm reference filter) using a SPECTROstar Nano plate reader (BMG LABTECH, Victoria, Australia). All samples were run in duplicate and samples from the same individual were run on the same plate. The sensitivity of the assay was 1.0 pg ml^−1^. The inter-assay coefficients of variation (CVs) for low and high controls were 5.52% and 12.98%, respectively (*n* = 3 plates), and the intra-assay CVs were 2.32% and 0.28%, respectively (*n* = 6 replicates).

To account for variation in urine concentration, melatonin–sulfate concentrations are expressed as nanograms per millilitre (ng ml^−1^) creatinine. Creatinine was determined using a modified Jaffe reaction (described in [46]). Briefly, 100 µl of standard, control or diluted urine sample was added to a 96-well microtiter plate, followed by 50 µl NaOH (0.75 M) and 50 µl picric acid (0.04 M). Plates were incubated for 15 min and read at 405 nm using a SPECTROstar Nano plate reader. The inter-assay CVs for low and high controls were 0.19% and 3.34%, respectively (*n* = 3 plates), and the intra-assay CVs were 0.97% and 1.72% respectively (*n* = 6 replicates).

### 2.6. Statistical analysis

All data were analysed using R v. 4.1.0 [47]. Preliminary analysis indicated the baseline phase and date of sample collection were confounded because all baseline samples were collected on the same night. Therefore, we excluded the baseline phase and restricted subsequent statistical analysis to the LED light treatments. Due to challenges in collecting urine samples and infrequent urination rates we did not have a sufficient distribution of samples to investigate the effect of time on melatonin–sulfate concentrations. Consequently, reported measures are less sensitive to time effects.

To examine the effect of light treatment (red, amber, warm white, cool white) on melatonin–sulfate concentrations, we fitted a linear mixed-effects model using R package lme4 [48] with Satterthwaite approximations for degrees of freedom (R package lmerTest [49]). Melatonin–sulfate concentration was modelled as a function of light treatment, sex and date of sample collection. Animal ID was included as a random effect to account for repeated samples from individuals under different light treatments. Model assumptions of normality and heterogeneity of variance were checked. Melatonin–sulfate concentrations were log-transformed prior to analysis to meet model assumptions.

## 3. Results

Melatonin–sulfate did not vary significantly in response to experimental light treatment (*F*_3,29.42_ = 0.32, *p* = 0.81), sample collection date (*F*_2,27.85_ = 1.18, *p* = 0.32) or animal sex (*F*_1,13.76_ = 1.19, *p* = 0.29).

**Figure 2.**
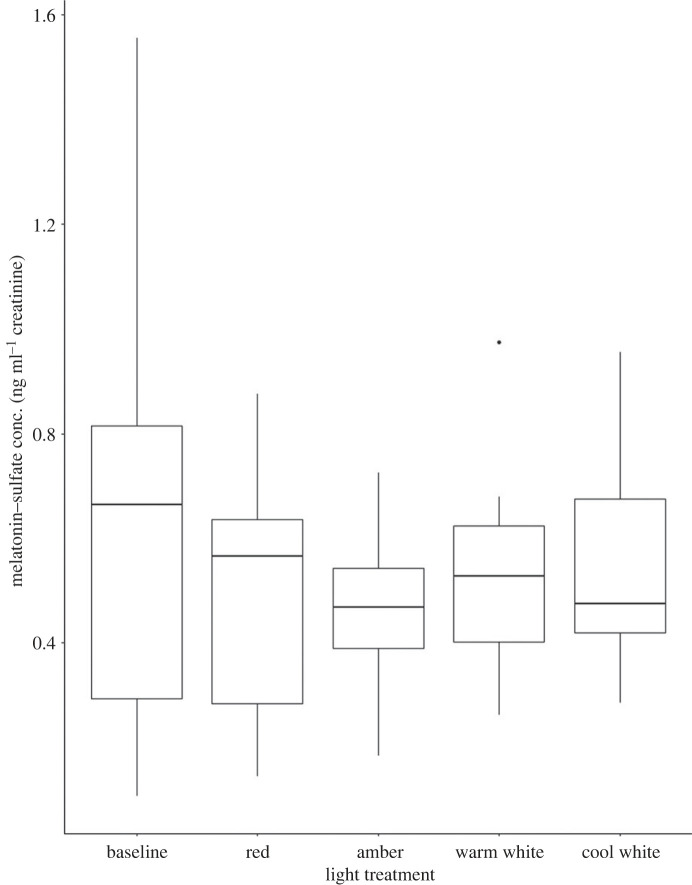
Urinary melatonin-sulfate concentrations (ng ml^−1^ creatinine) in Gould's wattled bats (*Chalinolobus gouldii)* exposed to ambient night-time light (baseline; *n* = 8) and four LED light treatments: red (*n* = 9), amber (*n* = 9), filtered warm white (*n* = 12) and cool white (*n* = 11).

Melatonin–sulfate concentrations ranged from 0.11 to 1.56 ng ml^−1^ creatinine (figure 2). The highest melatonin–sulfate concentrations (mean ± SEM ng ml^−1^ creatinine) were observed under ambient night-time conditions (baseline; 0.65 ± 0.161) followed by cool white LEDs (0.545 ± 0.065), filtered warm white (0.0531 ± 0.056), red (0.501 ± 0.08) and amber LEDs (0.468 ± 0.052). Mean melatonin–sulfate concentrations in males and females were 0.549 ± 0.072 (*n* = 20 samples) and 0.528 ± 0.038 (*n* = 29 samples), respectively.

## 4. Discussion

To our knowledge, this is the first study investigating the effect of artificial light at night on melatonin production in an insectivorous bat. The highest melatonin–sulfate concentrations were observed in Gould's wattled bats under ambient night-time conditions (baseline), while concentrations in bats under experimental LEDs were within the baseline range. We did not detect an effect of short-term exposure to light at night on melatonin concentrations in light-exploiting Gould's wattled bats. These findings support previous behavioural studies demonstrating Gould's wattled bats are not deterred by artificial light at night and may exploit the high abundance of insects at light sources [40].

Melatonin production is regulated by natural light-dark cycles. The photoreceptors that entrain melatonin rhythms are maximally sensitive to short-wavelength blue light (459–480 nm) [6–9]. The cool white LEDs used in this study contain a high proportion of blue wavelengths (range 420–780 nm, *λ*P 457 nm), while the filtered warm white LEDs (range 420–780 nm, *λ*P 586 nm) contain a small proportion, and the amber (range 500–780 nm, *λ*P 601 nm) and red LEDs (range 580–700 nm, *λ*P 630 nm) do not contain any blue wavelengths. Despite this, we did not find any correlation between the proportion of blue wavelengths and degree of melatonin suppression. Our findings contrast previous studies in mammals showing wavelength-dependent response of melatonin suppression in Syrian hamsters [16], social voles [15] and wallabies [17].

Duration of exposure plays a role in melatonin response to lights [15]. Given our study was limited to one night exposure to each spectral treatment, it is possible that a longer duration would yield different results, especially in a light-tolerant species which commonly roosts in urban environments and forages around light sources [40]. Additionally, we used ambient night-time for baseline measures opposed to complete darkness used in many laboratory-based studies. Ambient night-time provides a more ecologically relevant measure of baseline melatonin [50], this may explain differences between the current and previous studies. Furthermore, our study used voided urine samples resulting in a large sampling window (3.9 h). Consequently, observed melatonin–sulfate concentrations could be increasing, at the peak, or declining at the time of sampling [51]. Urinary melatonin metabolites have a lower concentration than plasma melatonin resulting in less resolution, especially for low concentrations [45], possibly explaining differences between our study and previous research [51].

We used light intensities representative of industry standards for lighting in Australian urban parklands (AS/NZS 11:58.3.1:2020) where our study species is often found [44]. In the wild, light-exploiting species, including Gould's wattled bats, spend short bouts of time in close proximity to light sources while foraging and would be exposed to a short duration of high intensity light. Both spectra and intensity play a role in regulating melatonin rhythms [6,18]. Future studies should investigate the impact of ecologically relevant light pulses of varying intensity on melatonin in insectivorous bats. Such studies will be particularly important to identify the impacts of short-wavelength light on physiology. Short-wavelengths entrain circadian physiology and are more attractive to nocturnal aerial invertebrates [52–55]. Light exploiting species are likely to spend more time foraging at short-wavelength light sources. Future studies should also investigate the effect of long-term light exposure on circadian physiology and activity patterns, representing permanent light installations in urban areas.

Considering the effect of artificial light on circadian physiology is critical in evaluating the efficacy of different lighting technologies to minimize disturbance to wildlife. Melatonin is a circadian hormone responsible for communicating photoperiod information to the body, leading to adaptive changes in endocrinology, physiology and anatomy [1,11]. Disruptions to the natural rhythm of melatonin, including from artificial light, can disrupt an animal's ability to adapt to environmental conditions. Suppressed melatonin can disrupt the timing of seasonal reproduction [42,56] with consequences for fitness and survival of species. Although we did not find evidence that short duration exposure to LEDs at night disrupts melatonin production in Gould's wattled bats, future research is required to investigate circadian physiology under ecologically relevant light regimes by manipulating the duration of exposure and light intensity. Long-wavelength LEDs should be tested on light-tolerant and light-averse species to evaluate their effectiveness as ‘bat friendly’ lighting. Understanding the effect of LEDs with different spectra and intensity on behaviour and physiology will be critical in determining appropriate lighting in wildlife sensitive areas.

## Data Availability

The dataset on which this article is based is available in the electronic supplementary material [57].
